# ID 215 - Painel de Preços da Saúde: uma tecnologia em saúde essencial para pesquisa de preços

**DOI:** 10.5327/2237-9622.2025.v34s1.215

**Published:** 2025-11-25

**Authors:** Carla de Paula Bernardes, Andressa Souza de Oliveira, Rejane Gabriela Silva, Thiago Mendonça Chagas

**Keywords:** Banco de Preços em Saúde, tecnologia, pesquisa de preço, licitação

## Abstract

**Introdução:**

O Banco de Preços em Saúde (BPS) é destinado ao registro e à consulta de informações de compras de medicamentos e produtos para a saúde realizadas por instituições públicas e privadas. Esse sistema foi desenvolvido pelo Ministério da Saúde e atualmente é gerenciado pela Coordenação de Acompanhamento e Qualificação da Gestão de Preço em Saúde, do Departamento de Economia e Desenvolvimento em Saúde, da Secretaria de Ciência, Tecnologia e Inovação e do Complexo Econômico-Industrial da Saúde. Os dados cadastrados no BPS podem ser utilizados pela União, pelos estados, pelo Distrito Federal e pelos municípios, com o objetivo de obter informações estatísticas, econômicas, socioeconômicos, epidemiológicos. Essas informações visam auxiliar na redução de dispêndios e possibilitar economicidade e melhor gestão dos recursos públicos. Alinhado ao BPS e à interoperabilidade de sistemas, em 2024 foi desenvolvido o Painel de Preço da Saúde (PPS), tecnologia essa que disponibiliza os dados inseridos no BPS para pesquisa de preços de forma rápida e precisa. O objetivo dessa ferramenta é disponibilizar aos gestores um banco de dados de fácil acesso para pesquisa de preços de medicamentos e produtos para saúde em todo o território nacional.

**Método:**

Estudo descritivo, do tipo relato de experiência.

**Resultados:**

O PPS possui uma linguagem simples e acessível, facilitando sua usabilidade em estudos econômicos. Os filtros estão relacionados aos dados: período da compra, código do material conforme a descrição do Catálogo de Materiais (Catmat), nome do fornecedor do item, unidade de fornecimento, modalidade da compra. Os resultados das buscas podem ser apresentados em forma de tabelas e gráficos.

**Conclusão:**

O PPS é considerado uma tecnologia em saúde essencial, pois contribui e subsidia os gestores em seus processos licitatórios, permitindo um melhor poder de negociação dos gestores do SUS, e possibilita aquisições de medicamentos e produtos para a saúde em consonância aos melhores preços praticados no mercado e melhor alocação e transparência quanto à utilização dos recursos públicos. No momento em que as compras dos entes federados são registradas no sistema, as informações se tornam públicas, disponíveis para consulta, passando a ser referência para a pesquisa de preços. Além disso, permite qualificar a pesquisa de preços no âmbito do processo licitatório (informações regionalizadas, tratamento estatístico das informações de preço, comparação entre preços praticados, preços regulados, grau de concentração de mercado por princípio ativo, entre outros), o acompanhamento do histórico de compras e a evolução dos preços praticados pela instituição compradora. A pesquisa de preços é uma das etapas que antecede ao processo licitatório, e o PPS contribui com embasamento para comparar e examinar as propostas recebidas. Por esse motivo, a estimativa de preços é crucial e necessária para a contratação na Administração Pública, por meio da indicação de preços disponibilizados nos certames licitatórios. Conclusão: essa ferramenta auxilia na identificação de variações de preço, na avaliação de competitividade do mercado e na realização de análise de tendências e padrões de consumo.

